# Hydration, Hyperthermia, Glycogen, and Recovery: Crucial Factors in Exercise Performance—A Systematic Review and Meta-Analysis

**DOI:** 10.3390/nu15204442

**Published:** 2023-10-19

**Authors:** Olga López-Torres, Celia Rodríguez-Longobardo, Rodrigo Escribano-Tabernero, Valentín E. Fernández-Elías

**Affiliations:** 1 Sports Department, Faculty of Physical Activity and Sport Sciences, Universidad Europea de Madrid, 28670 Madrid, Spain; escribanorodrigo@gmail.com (R.E.-T.); valentin.fernandez@universidadeuropea.es (V.E.F.-E.); 2 Social Sciences of Physical Activity, Sport and Leisure Department, Faculty of Physical Activity and Sport Sciences, Universidad Politécnica de Madrid, 28040 Madrid, Spain; celia.rlongobardo@upm.es

**Keywords:** dehydration, muscle temperature, athletic performance, glycogen recovery

## Abstract

Hyperthermia accelerates dehydration and can lead to a glycolysis malfunction. Therefore, to deeply understand the relationship between dehydration and hyperthermia during exercise, as well as in the recovery time, there might be important factors to improve athletic performance. A systematic review was carried out in different databases using the words “hydration” OR “dehydration” AND “glycogen” OR “glycogenesis” OR “glycogenolysis” AND “muscle” OR “muscle metabolism” OR “cardiovascular system” and adding them to the “topic section” in Web of Science, to “Title/Abstract” in PubMed and to “Abstract” in SPORTDiscus. A total of 18 studies were included in the review and 13 in the meta-analysis. The free statistical software Jamovi was used to run the meta-analysis (version 1.6.15). A total sample of 158 people was included in the qualitative analysis, with a mean age of 23.5 years. Ten studies compared muscle glycogen content after hydration vs. remaining dehydrated (SMD −4.77 to 3.71, positive 80% of estimates, \hat{\mu} = 0.79 (95% CI: −0.54 to 2.12), z = 1.17, *p* = 0.24, Q-test (Q(9) = 66.38, *p* < 0.0001, tau^2^ = 4.14, *I*^2^ = 91.88%). Four studies examined the effect of temperature on postexercise muscle glycogen content (SMD −3.14 to −0.63, 100% of estimates being negative, \hat{\mu} = −1.52 (95% CI: −2.52 to −0.53), (z = −3.00, *p* = 0.003, Q-test (Q(3) = 8.40, *p* = 0.038, tau^2^ = 0.68, *I*^2^ = 66.81%). In conclusion, both hyperthermia and dehydration may contribute to elevated glycogenolysis during exercise and poor glycogen resynthesis during recovery. Although core and muscle hyperthermia are the key factors in glycogen impairments, they are also directly related to dehydration.

## 1. Introduction

Water is a fundamental component of the human body (~70% of body composition) and plays a crucial role in numerous physiological processes. In the muscular tissue, water participates in several processes related to muscle contraction, such as ATP hydrolysis in the myosin heads and chemical reactions in the Krebs cycle and in the final step of the electron transport chain in the mitochondria [1]. Regarding glycogen metabolism, it has been accepted that glycogen is stored in the muscle linked to at least 3 g of water [2,3]. Moreover, with respect to glycogenesis and glycolysis, hydration status plays a key role in both, both during and after exercise [4,5]. As bodily fluids are lost during exercise due to sweating, water content in the muscular cells decreases. After exercise, in order to maintain cardiovascular function stability, the body prioritizes the restoration of the plasma volume. Therefore, if rehydration during [6,7] or after [8] exercise is not complete, muscular tissue hydration can be compromised [9,10]. At the same time, based on animal models, it can be hypothesized that muscular water deficit may lead to an increase in the use of muscular glycogen in humans [11,12].

Another determinant factor in glycogen metabolism is the increase in temperature in active musculature during exercise. It has been demonstrated that exercising in a hot environment (41 °C) increased the utilization of intramuscular glycogen compared to exercise in a cold environment (9 °C) [13,14]. Subsequently, hyperthermia in active muscles during exercise increases glycogen expenditure [4,15,16].

After prolonged exercise, glycogenesis begins in depleted muscles. If sufficient carbohydrates (CHO) are ingested (i.e., 1–1.2 g/kg/h), 40% of glycogen levels can be restored within the first hour of recovery [17,18] and around 75% within 4–5 h postexercise [19]. The total restorage will be completed within about 24 h [19]. A similar event occurs with muscular hydration restorage, which will be totally restored in the 4 h after exercise if sufficient liquid is ingested (1.5 L of the total body weight lost during exercise) [4]. Therefore, both glycogen and muscular water lost due to exercise are restored in parallel within the early hours of recovery when enough water and CHO are consumed. Studies carried out to analyze the interaction between hydration level and glycogen restorage showed that in the short run (4 h postexercise recovery), subjects who remained dehydrated had reduced glycogen stored in a manner close to being significant [3]. On the other hand, in the long run (15 h postexercise recovery), water deprivation did not hinder normal glycogen resynthesis [20].

Fernandez-Elias, Ortega, et al. [4] demonstrated that in postexercise recovery with adequate CHO intake, participants who limited water consumption showed a ratio of 1 g of glycogen to 3 g of water in the analyzed muscle tissue. However, in participants who, in addition to CHO, ingested an amount of water equal to the loss during exercise, a ratio of 1 g of glycogen to 17 g of water was observed.

Despite the relevance of this topic, few studies have been investigated in the past years. Technology and resources that have been improved in the last decade might help with the biochemical and molecular mechanisms that occur inside the myocyte to better understand the events in the cell induced by hyperthermia and dehydration.

Therefore, to deeply understand the role of rehydration and CHO ingestion after exercise, as well as hyperthermia, it might help to optimize training and recovery strategies.

## 2. Methodology

### 2.1. Search Strategy

A systematic review of the literature and meta-analysis was conducted following the Preferred Reporting Items for Systematic Reviews and Meta-Analyses (PRISMA) guidelines and the patient, problem or population, intervention, comparison, control or comparator, outcome(s), and study PICOS criteria (Population: recreationally active or trained males and females; Intervention: glycogen utilization after a performance test; Comparison, control or comparator: standard conditions or hyperthermia and/or dehydration; Outcome: optimize training and recovery strategies; Study type: crossover interventions).

A literature search was conducted using PubMed, Web of Science and SPORTDiscus databases, selecting articles published up to February 2023. A search strategy was carried out using the words “hydration” OR “dehydration” AND “glycogen” OR “glycogenesis” OR “glycogenolysis” AND “muscle” OR “muscle metabolism” OR “cardiovascular system” and adding them to the “topic section” in Web of Science, to “Title/Abstract” in PubMed and to “Abstract” in SPORTDiscus.

The inclusion criteria: articles that analyzed the effects of muscle hydration on glycogen metabolism and the effects of dehydration on the cardiovascular system, that were experimental in design, and any language and date of publication.

Exclusion criteria: studies conducted on animals, children (<18 years old), the elderly (>65 years old), physically inactive people, people with pathologies or clinical conditions, and studies in which the effect of muscle hydration on glycogen metabolism or the cardiovascular system was not directly determined.

**Figure 1:**
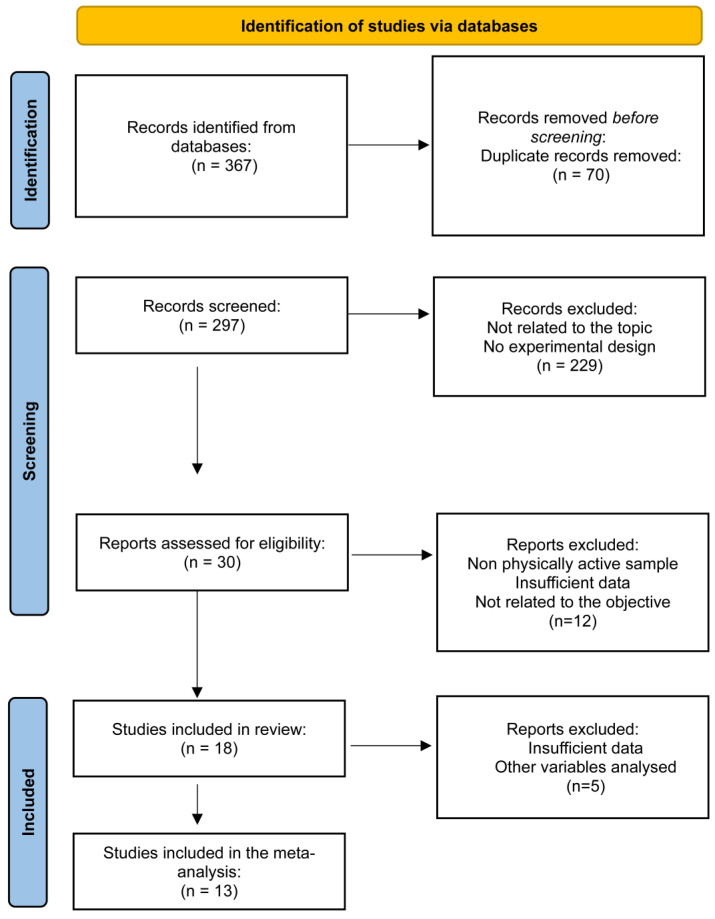
Flowchart for study selection.

Originally, 367 items were identified. After removing duplicates, nonexperimental articles, and those that did not meet the inclusion criteria, 18 studies were included in the systematic review and 13 in the meta-analysis. A flowchart of study selection is shown in Figure 1. Errata regarding the studies included were checked. One study included had an editorial letter, which was reviewed. The comments did not affect the data used in the present systematic review and meta-analysis.

### 2.2. Data Extraction

Two independent authors extracted the following variables from each article: author, year and journal, title, objective, sample, exercise test carried out, and conclusions. If any disagreement appeared, a third author resolved them.

The main outcome analyzed was the glycogen muscle utilization after exercise. Data from two possible situations were collected: after fluid intake (hydration) compared to dehydration or glycogen content after exercising in the heat compared to exercising in a neutral temperature.

### 2.3. Risk of Bias

Two independent reviewers analyzed the risk of bias assessment with the PEDro scale [21]. This tool rates the studies on a scale from 0 to 10, where a score of 0–3 ranks as ‘poor’, 4–5 is ‘fair’, 6–8 ‘good’, and 9–10 ‘excellent’. It gives information about the randomization and allocation process of the articles, the blinding of subjects, therapists, and assessors, the measure of the outcomes, and the statistical analysis.

### 2.4. Data Analysis

The analysis was conducted using the standardized mean difference (SMD) as the outcome measure. A random-effects model was fitted to the data. The amount of heterogeneity (i.e., tau^2^) was estimated using the restricted maximum-likelihood estimator [22]. In addition to the estimate of tau^2^, the Q-test for heterogeneity [23] and the *I*^2^ statistic were shown. In case any amount of heterogeneity was detected (i.e., tau^2^ > 0, regardless of the results of the Q-test), a prediction interval for the true outcomes was also given. To examine whether studies may be outliers and/or influential in the context of the model, studentized residuals and Cook’s distances were used. Studies with a studentized residual larger than the 100 × (1–0.05/(2 × k))th percentile of a standard normal distribution were considered potential outliers (i.e., using a Bonferroni correction with two-sided alpha = 0.05 for k studies included in the meta-analysis). Studies with a Cook’s distance greater than the median plus six times the interquartile range of the Cook’s distances were considered influential. To check for funnel plot asymmetry, the rank correlation test and the regression test were used, applying the standard error of the observed outcomes as a predictor. The free statistical software Jamovi was used to run the analysis (version 1.6.15) [24].

## 3. Results

The selected articles analyzed the effect of dehydration on cardiovascular parameters and glycogen metabolism due to a lack of fluid intake at a stable environmental temperature and/or due to exercising at high temperatures.

Articles included in the systematic review dated from 1976 to 2017. A total sample of 158 people was included in the qualitative analysis, with a mean age of 23.5 years. Of the 18 articles selected, nine involved endurance-trained males (50%, *n* = 86) [3,4,9,10,13,14,15,17,25], five engaged recreationally active males (27.8% *n* = 40) [16,18,20,26,27], two included male ice-hockey players (11.1%, *n* = 15) [28,29], one was carried out with international class lightweight rowers (5.6%, *n* = 8) [30], and another one with recreationally active females (5.6% *n* = 9) [31]. Eight of the 18 articles (44.4%) used a muscle biopsy to extract glycogen content, while nine studies (50%) also added blood samples to collect the data. The three remaining studies used blood samples alone, blood and urine samples, or urine and muscle biopsy samples, respectively. Eleven articles [9,10,15,17,18,20,26,28,29,30,31] analyzed the effect of fluid intake vs. staying dehydrated on glycogen utilization after exercising in environmental temperatures, while six [3,13,14,16,25,27] studies analyzed the effects of exercising in the heat on the same aspect. One article compared both of the above aspects [4] (Table 1).

### 3.1. Quantitative Analysis

#### 3.1.1. Rehydration vs. Dehydration

**Figure 2:**
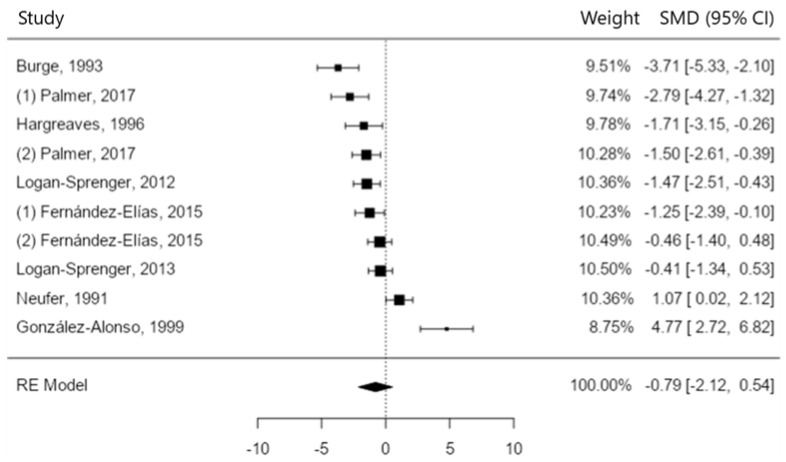
Muscle glycogen resynthesis after exercise [3,4,15,20,25,26,28,29,30,31].

Ten studies comparing the muscle glycogen content after hydration vs. remaining dehydrated postexercise were included in the analysis [3,4,15,20,25,26,28,29,30,31] (see Figure 2). The observed SMD varied from −4.77 to 3.71, being positive in the majority of estimates (80%). The average SMD based on the random-effects model was \hat{\mu} = 0.79 (95% CI: −0.54 to 2.12). Thus, the average outcome was not significantly different from zero (z = 1.17, *p* = 0.24). The true outcomes appear to be heterogeneous according to the Q-test (Q(9) = 66.38, *p* < 0.0001, tau^2^ = 4.14, *I*^2^ = 91.88%). A 95% prediction interval for the true outcomes is given from −3.41 to 4.99. Therefore, even though the average outcome is positive, in some studies, the true outcome may, in fact, be negative. An exploration of the studentized residuals showed that one study [25] had a value larger than ± 2.81 and could be a potential outlier in the context of this model. None of the studies could be considered to be overly influential according to the Cook’s distances. Neither the rank correlation nor the regression test indicated any funnel plot asymmetry (*p* = 0.381 and *p* = 0.396, respectively).

#### 3.1.2. High Temperature vs. Low Temperature

**Figure 3:**
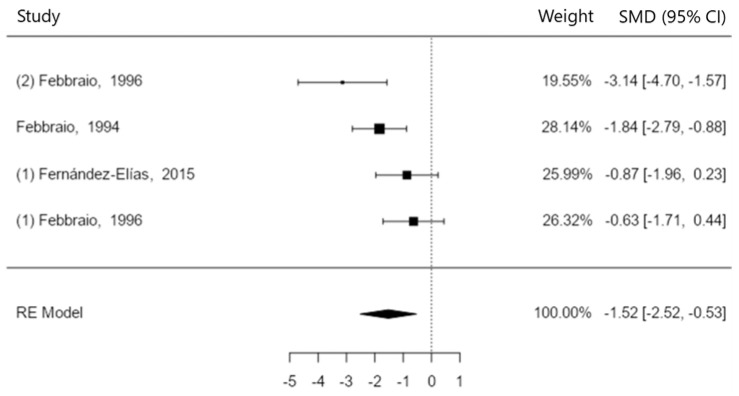
Muscle glycogen utilization after exercising in the heat [4,13,14,16].

Four studies that examined the effect of temperature on postexercise muscle glycogen content were added to this analysis [4,13,14,16] (see Figure 3). The observed SMD ranged from −3.14 to −0.63, with all of estimates being negative (100%). The estimated average SMD according to the random-effects model was \hat{\mu} = −1.52 (95% CI: −2.52 to −0.53). Hence, the average outcome differed significantly from zero (z = −3.00, *p* = 0.003). Based on the Q-test, the true outcomes seem to be heterogeneous (Q(3) = 8.40, *p* = 0.038, tau^2^ = 0.68, *I*^2^ = 66.81%). A 95% prediction interval for the true outcomes ranged from −3.41 to 0.37. Thus, although the average outcome is considered to be negative, in some studies, the true outcome could, in reality, be positive. An exploration of the studentized residuals showed that none of the studies had a value larger than ± 2.50, and therefore, there was no indication of outliers in this model. Taking into account Cook’s distances, none of the studies could be contemplated to be overly influential. Neither the rank correlation nor the regression test indicated any funnel plot asymmetry (*p* = 0.750 and *p* = 0.176, respectively). Hence, the results indicated that exercising in hot conditions (whether internal in the muscle fiber or external due to climate) decreases glycogen utilization when comparing the same exercise intensities (one at neutral temperature and another one at high temperature).

## 4. Risk of Bias Assessment

According to the PEDro tool, 10 out of the 13 articles received a score of 4 to 5, considered as ‘fair’ [3,4,13,14,15,26,28,29,30,31]. The three remaining articles scored a 3, with this rating as ‘poor’ [16,20,25] (Table 2).

There were three articles that did not specify if the allocations to treatments were random. [15,20,25] No blinding process was carried out in any study. Only two articles explicitly specified the initial number of participants and the final number from which the results were obtained. [13,15] Fernández-Elías et al. were the only authors who provided effect sizes in their studies. [3,4].

## 5. Discussion

The present systematic review and meta-analysis focus on analyzing the interaction between temperature and hydration status regarding glycogen utilization during exercise, with a specific emphasis on glycogen restoration and resynthesis after exercise. Maintaining high and stable levels of muscular glycogen during prolonged or high-intensity exercise is crucial for sports performance. The results from this systematic review demonstrate that both hydration status and intramuscular temperature play key roles in glycogenolysis and glycogen use during exercise, as well as in glycogen resynthesis during the recovery period.

During exercise, especially in long-duration or high-intensity activities, intramuscular, and thus, body temperature, rises considerably. When exercising in hot environments, body heating increases more rapidly and to a greater extent. To maintain body temperature, sweat is produced and, therefore, body fluids decrease, causing dehydration. When body fluids are not completely restored, the central temperature rises, and body fluid losses [32] result in an alteration of glycogen use [4]. However, the physiological mechanism behind this is not fully understood yet.

Regarding the level of hydration, the body’s level of hydration is primarily influenced by the balance between fluid intake and fluid loss. The quantity of blood in the body, although vital for overall health and bodily functions, does not directly determine one’s hydration status. During exercise, especially in hot conditions, when fluid intake is insufficient and dehydration occurs, plasma volume can decrease [9]. It is worth noting that this reduction in plasma volume does not significantly impact active muscles. As a result, the hydration level of active muscles remains relatively stable, even when the overall body is becoming dehydrated. [10] Therefore, the decrease in blood volume does not directly affect muscle metabolism during exercise.

Some studies analyzed in the present systematic review concluded that hyperthermia related to exercise is the main and most crucial factor that influences glycogen metabolism and, hence, performance. Gonzalez-Alonso et al. [25] examined whether reductions in muscle blood flow due to dehydration would reduce substrate delivery and metabolite exchange to and from active skeletal muscles during prolonged exercise in the heat. The authors found that hyperthermia and not blood flow reduction was the main factor underlying the early fatigue in prolonged cycling. Other studies showed the same conclusions [4,31].

Nevertheless, maintaining a correct fluid intake during exercise can help reduce temperature increase [32]. Therefore, although maintaining body core and intramuscular temperature below 40 °C [1] is the crucial factor to maintain optimal use of glycogen, ingesting an adequate amount of fluid during exercise would help prevent hyperthermia and contribute to maintaining glycogen metabolism efficiency. This fact is even more crucial when exercising in hot environments since it will increase body fluid losses through sweating and, thus, trigger a glycogen utilization impairment [4].

Due to the contribution of fluid intake to temperature control during exercise, many studies included in the present systematic review focused on analyzing how hydration or dehydration statuses influence glycogen use during and after exercise. In general, authors [3,4,15,26,28,29,30,31] agree that dehydration accelerates the use of glycogen compared to similar exercise intensities. The study of Logan-Sprenger et al. [31] in recreationally active females concluded that progressive dehydration significantly increased heart rate, core temperature, rate of perceived exertion, whole body carbohydrates oxidation, and muscle glycogenolysis. Moreover, the increased muscle glycogenolysis related to dehydration appeared to be due to increased core and muscle temperature. Other studies [15,30] showed the same conclusions. On the contrary, another study carried out by the same authors but in recreational men found the same results except for carbohydrate oxidation [26]. In this study, the male sample did not show a carbohydrate oxidation reduction with dehydration.

Thus, the results from the meta-analysis of the included studies indicated that insufficient or no fluid ingestion after exercise decreases glycogen restoration (or resynthesis), which can derive a lower glycogen utilization in the next bout of exercise.

Finally, when those factors were analyzed during exercise recovery, most of the studies concluded that adequate fluid replacement after prolonged or intense exercise can help optimally restore muscular glycogen. In both studies performed by Fernandez-Elias et al. [3] and Neufer et al. [20], the results agreed that even with poor or no liquid replacement after exercise, muscular glycogen resynthesis occurred. Neufer et al. [20] concluded that despite reduced water content during the first 15 h of recovery, muscle glycogen resynthesis is not impaired. In the same direction is the study of Fernández-Elías et al. [3]. These authors found that dehydration in the recovery did not impair muscle glycogen storage (glycogen/water gs ratio 1/3). Nevertheless, if fluid ingestion occurs properly, glycogen restoration is more optimal (glycogen/water gs ratio 1/17). Hence, even if the amount of ingested carbohydrates is enough during the recovery time, if fluid replacement is low and dehydration continuous, glycogen replacement may be impaired. Therefore, a poor postexercise rehydration strategy may lead to a hypohydrated status, which, in turn, may result in an increased glycogen utilization rate during the subsequent exercise session. This can decrease performance, especially in disciplines where high-intensity performance is required over consecutive days.

This systematic review has some limitations, as some of the studies included in it are from the past century. Initial studies carried out during the 1980s and 1990s drew conclusions that have not been retested. Some authors continued the research in this field in the past years, but new studies using new available technologies are needed to confirm the current statements and deepen into new conclusions. Regarding the risk of bias in the studies, although there are some parameters that are difficult to carry out, such as the blinding process, since the subjects are aware of whether they are hydrating or not or of the temperature conditions, all the studies obtained a rather low score, so the results presented should be taken with caution.

## 6. Conclusions

Both hyperthermia and dehydration may contribute to elevated glycogenolysis during exercise and poorer glycogen resynthesis during recovery. While core and muscle hyperthermia, resulting from high-intensity or prolonged exercise (internal hyperthermia) or exercising in hot environments (external hyperthermia), appears to be a significant factor contributing to glycogen impairments, it is closely linked to dehydration. Maintaining an appropriate body temperature and ensuring proper fluid intake during and after exercise are essential for optimizing long-term performance. As the practical applications seem to be clear, more studies are needed to deepen the biomechanical aspects that help understand the influence of hyperthermia and dehydration on glycogen metabolism.

## Figures and Tables

**Table 1:** Study characteristics included in the systematic review.

Author, Year-Journal	Title	Aim	Sample (*n*), Age	Data Extraction	Exercise	Conclusions
Costill, 1976—J Appl Physiol [27]	Muscle water and electrolytes following varied levels of dehydration in men	To describe (1) the effects of varied levels of dehydration on muscle water and electrolytes and (2) the relationship between plasma and muscle water and electrolytes following large sweat losses	Healthy men (8), 28.0	Blood urine and muscle biopsy samples	3 × 1.5 h cycling at 70% VO_2max_ at 39.5 °C (25% relative humidity) with 30’ rest between trials to reduce 2% BW in each trial	Body water lost during exercise in the heat is attributed to relatively larger water losses from extracellular than from intracellular compartments. However, in terms of absolute losses, both intra- and extracellular volumes contribute similar quantities of water to account for the total fluid losses
Hamouti, 2013—Eur J Appl Physiol [9]	Comparison between blood and urinary fluid balance indices during dehydrating exercise and the subsequent hypohydration when fluid is not restored	To determine the ability of urine-specific gravity (U_SG_) to detect the low levels of dehydration (2% of body mass loss) induced during the early stages of prolonged exercise in the heat. Additionally, we sought to determine if U_SG_ reflects long-term (i.e., 11 h) body fluid deficit better than blood serum osmolality (S_OSM_).	Aerobically trained male athletes (18), 20.3 ± 1.9	Urine and blood samples	Cycling for bouts of 20 min at a 60% VO_2max_ interspersed with 10 min of rest until they lost 3% of their initial body mass	When comparing 3% dehydration (end of exercise) to 3% hypohydration (next morning), U_SG_ increased (1.025 ± 0.003 to 1.028 ± 0.003; *p* < 0.05) while S_OSM_ decreased (295 ± 5 to 287 ± 5 mO-smol kg^−1^H2O; *p* < 0.05)
Mora-Rodríguez, 2015—Scand J Med Sci Sports [10]	Skeletal muscle water and electrolytes following prolonged dehydrating exercise	To study if dehydrating exercise would reduce muscle water (H_2_O_muscle)_ and affect muscle electrolyte concentrations.	Endurance-trained male cyclists (9), 24 ± 9	Muscle biopsy	Cycling at 65% VO_2max_ during 120 min followed by 30 more minutes at 55% VO_2max_ (33 ± 1 °C, 25% ± 2% humidity)	After 4 h of recovery, PV returned to pre-exercise values; however, H_2_O_muscle_ remained reduced at the same level. Muscle Na^+^ and K^+^ increased (*p* < 0.05) in response to the H_2_Omuscle reductions
Hargreaves, 1996 J Appl Physiol [15]	Effect of fluid ingestion on muscle metabolism during prolonged exercise	To examine the effect of fluid ingestion on muscle metabolism	Trained men (5), 27 ± 2.8	Muscle biopsy and blood sample	Cycling 2 h at 67% VO_2max_ in a 20–22 °C environment with or without fluid ingestion (FR vs. NF, respectively)	Fluid ingestion reduces muscle glycogen use during prolonged exercise, which may account, in part, for the improved performance previously observed with fluid ingestion
Logan-Sprenger, 2012—Med Sci Sports Exerc [31]	Effects of dehydration during cycling on skeletal muscle metabolism in females	To investigate the effects of progressive dehydration on the time course of changes to whole body substrate oxidation and skeletal muscle metabolism during 120 min of cycling in hydrated females	Recreationally active females (9), 21.7 ± 0.6	Muscle biopsy and blood sample	120 min cycling at 65% VO_2max_ on two occasions: with no fluid (DEH) and with fluid (HYD) replacement	Progressive dehydration significantly increased HR, Tc, RPE, Pvol loss, whole body CHO oxidation, and muscle glycogenolysis. The increased muscle glycogenolysis with DEH appeared to be due to increased core and muscle temperature, secondary to less efficient movement of heat from the core to the periphery
Logan-Sprenger, 2013—Int J Sport Nutr Exerc Metab [26]	Increase in skeletal muscle glycogenolysis and perceived exertion with progressive dehydration during cycling in hydrated men	To investigate the effects of progressive mild dehydration during cycling on whole-body substrate oxidation and skeletal muscle metabolism in recreationally active men	Recreationally active men (9), 21.6 ± 20.5	Muscle biopsy and blood sample	120 min cycling at 65% VO_2max_ on two occasions: with no fluid (DEH) and with fluid (HYD) replacement	Dehydration of < 2% BM elevated physiological parameters and perceived exertion, as well as muscle glycogenolysis, during exercise without affecting whole-body CHO oxidation
Fernández-Elías, 2015—Scand J Med Sci Sports [4]	Hyperthermia, but not muscle water deficit, increases glycogen use during intense exercise	To determine if dehydration alone or in combination with hyperthermia accelerates muscle glycogen use during intense exercise	Endurance-trained male cyclists (7), 22.0 ± 2.4	Muscle biopsy and blood sample	40 min at 75% VO_2max_ in a neutral (25 ± 1 °C) environment after dehydration trial (HYPO) or rehydration (REH) or 40 min at 75% VO_2max_ in a hot environment (36 ± 1 °C; REH_HOT_)	Hyperthermia stimulates glycogen use during intense exercise, while muscle water deficit has a minor role
Fernández-Elías, 2015—Eur J Appl Physiol [3]	Relationship between muscle water and glycogen recovery after prolonged exercise in the heat in humans	To investigate the role of rehydration in muscle glycogen recovery in subjects who underwent two trials with the same provision of carbohydrates but very different volumes of water ingested	Endurance-trained male cyclists (9), 24 ± 3	Muscle biopsy	150 min cycling at 65% VO_2max_ in a hot, dry environment (33 ± 4 °C). One hour after exercise, subjects ingested 250 g of carbohydrates in 400 mL of water (REH_LOW_) or the same syrup plus water to match fluid losses (3170 ± 190 mL; REH_FULL_)	Despite the insufficient water provided during REH_LOW_, per each gram of glycogen, 3 g of water was stored in muscle (recovery ratio 1:3), while during REH_FULL_, this ratio was higher (1:17)
Ivy, 1988—J Appl Physiol [18]	Muscle glycogen storage after different amounts of carbohydrate ingestion	To determine whether the rate of muscle glycogen storage could be enhanced during the initial 4-h period postexercise by substantially increasing the amount of the carbohydrate consumed	Recreational cyclists, men (8)	Muscle biopsy and blood sample	45 min cycling at 70–75% VO_2max_ followed by 10 min of high-intensity interval cycling. Immediately and 2 h after exercise, they consumed either 0 (P), 1.5 (L), or 3.0 g glucose/kg body wt (H) from a 50% glucose polymer solution	1.5 g glucose/kg body wt provided immediately and 2 h after exercise will significantly enhance muscle glycogen restoration above the basal rate. However, doubling the amount of the glucose supplement from 1.5 to 3.0 g/kg body wt is of no additional benefit regarding glycogen restoration during the initial hours after exercise
Ivy, 1988—J Appl Physiol [17]	Muscle glycogen synthesis after exercise: effect of time of carbohydrate ingestion	To examine the time of ingestion of a carbohydrate supplement on muscle glycogen storage postexercise	Male cyclists (12), 26.1 ± 5.1	Muscle biopsy and blood sample	70 min on a cycle ergometer at 68% VO_2max_, interrupted by six 2 min intervals at 88% VO_2max_, on two separate occasions: ingesting a 25% carbohydrate solution (2 g/kg body wt) immediately postexercise (P-EX) or 2 h postexercise (2P-EX)	Delaying the ingestion of a carbohydrate supplement postexercise will result in a reduced rate of muscle glycogen storage
Neufer, 1991—J Appl Physiol [20]	Hypohydration does not impair skeletal muscle glycogen resynthesis after exercise	To examine the effects of moderate hypohydration skeletal muscle glycogen resynthesis after exhaustive exercise.	Active males (8), 19 ± 1	Muscle biopsy	2 h of intermittent cycle ergometer exercise (4 bouts of 17 min at 60% and 3 min at 80% of VO_2max_/10 min rest) followed by several hours of light upper body exercise in the heat without fluid replacement (HY) or with water ingestion (EU)	Despite reduced water content during the first 15 h after heavy exercise, skeletal muscle glycogen resynthesis is not impaired
Palmer, 2017—Int J Sports Med [28]	Ingesting A Sports Drink Enhances Simulated Ice Hockey Performance While Reducing Perceived Effort	To determine whether ingesting a carbohydrate–electrolyte solution (CES) vs. progressive dehydration affects skeletal muscle glycogen use and performance in ice hockey players during simulated ice hockey exercise	Male ice-hockey players (7), 21.3 ± 0.3	Muscle biopsy	Three gameplay “periods” separated by 10-min intermissions Each period contained 10 high-intensity, intermittent cycling sprints to simulate gameplay. Each cycling sprint lasted 45 s at an average of 133 % VO_2max_, followed by 135 s of passive rest on the ergometer	Compared to progressive dehydration, staying hydrated by ingesting a CES helps preserve performance, while reducing thermal and perceptual strains, in the 3rd period of cycle-based simulation of ice hockey exercise. These benefits are observed despite greater glycogen use in the 3rd period with CES ingestion
Palmer, 2017—IJSNEM [29]	Mild Dehydration Does Not Influence Performance or Skeletal Muscle Metabolism During Simulated Ice Hockey Exercise in Men	To determine whether mild dehydration influences skeletal muscle glycogen use, core temperature, or performance during high-intensity, intermittent, cycle-based exercise in ice hockey players vs. staying hydrated with water	Male ice-hockey players (8), 21.6 ± 0.4	Muscle biopsy	Three periods (P) containing 10 × 45-s cycling bouts at 133% VO_2max_, followed by 135 s of passive rest while being dehydrated during the protocol (NF) or maintaining body mass by drinking water.	Typical dehydration experienced by ice hockey players (~1.8% BM loss) did not affect glycogen use, core temperature, or voluntary performance vs. staying hydrated by ingesting water during a cycle-based simulation of ice hockey exercise in a laboratory environment
Burge, 1993—Med Sci Sports Exerc [30]	Rowing performance, fluid balance, and metabolic function following dehydration and rehydration	To determine the efficacy of rehydrating with water following 24 h of dehydration on body fluid balance, metabolic function, and rowing performance	International class lightweight rowers (8)	Muscle biopsy	Maximal rowing trial on a Gjessing rowing ergometer (4200 revs, 3 kg resistance) while euhydrated (ET) and following partial rehydration (RT).	The dehydration/rehydration protocol reduced maximal rowing performance due to lowered plasma volume and decreased muscle glycogen utilization
González-Alonso, 1999—J Physiol [25]	Metabolic and thermodynamic responses to dehydration-induced reductions in muscle blood flow in exercising humans.	To examine whether reductions in muscle blood flow with exercise-induced dehydration would reduce substrate delivery and metabolite and heat removal to and from active skeletal muscles during prolonged exercise in the heat	Endurance-trained males (7), 27 ± 2	Muscle biopsy and blood sample	Cycling in the heat (35 °C; 61 ± 2% VO_2max_) dehydrated (DE) or ingesting fluids and stabilizing T (CON)	Hyperthermia, rather than altered metabolism, is the main factor underlying the early fatigue with dehydration during prolonged exercise in the heat
Febbraio, 1994—J Appl Physiol. [13]	Effect of heat stress on muscle energy metabolism during exercise.	To examine the effect of heat stress on muscle energy metabolism during submaximal exercise	Endurance-trained men (12), 21.6 ± 0.5	Muscle biopsy	Cycling 40 min at 70% VO_2max_ in 20 °C and 20% relative humidity (T_20_) or 40 °C and 20% relative humidity (T_40_).	Muscle glycogenolysis is increased in the heat
Febbraio, 1996—Exp Physiol [14]	Blunting the rise in body temperature reduces muscle glycogenolysis during exercise in humans.	To examine the effect of blunting the rise in body temperature on exercise metabolism	Endurance-trained men (7), 22.0 ± 2.4	Muscle biopsy and blood sample	Cycling 40 min at 65% VO_2max_ in 20 °C and 20% relative humidity (T_20_) or 3 °C and 50% relative humidity (T_3_).	When the rise in body core temperature is attenuated, the glycogenolysis in contracting skeletal muscle is reduced during exercise
Febbraio, 1996—Am J Physiol [16]	Influence of elevated muscle temperature on metabolism during intense, dynamic exercise	To examine the effects of elevated muscle temperature on muscle metabolism during exercise	Active but untrained men (7), 28.7 ± 7.0	Blood sample	Two min cycle ergometer trials at 115% VO_2max_ without pretreatment (CON) or after having their thigh wrapped in a heating blanket for 60’ before exercise (HT)	Net muscle glycogen use was higher in HT. An elevated T per se increases muscle glycogenolysis, glycolysis, and high-energy phosphate degradation during exercise

**Table 2:** PEDro risk of bias scores.

Study	Items										Total Score
	1	2	3	4	5	6	7	8	9	10	
Burge, 1993 [30]	🗴	🗴	🗴	🗴	🗴	🗴	🗴	🗴	🗴	🗴	4/10
Palmer, 2017 [28]	🗴	🗴	🗴	🗴	🗴	🗴	🗴	🗴	🗴	🗴	4/10
Palmer, 2017 [29]	🗴	🗴	🗴	🗴	🗴	🗴	🗴	🗴	🗴	🗴	4/10
Hargreaves, 1996 [15]	🗴	🗴	🗴	🗴	🗴	🗴	🗴	🗴	🗴	🗴	4/10
Logan-Sprenger, 2012 [31]	🗴	🗴	🗴	🗴	🗴	🗴	🗴	🗴	🗴	🗴	4/10
Fernández-Elías, 2015 [3]	🗴	🗴	🗴	🗴	🗴	🗴	🗴	🗴	🗴	🗴	5/10
Fernández-Elías, 2015 [4]	🗴	🗴	🗴	🗴	🗴	🗴	🗴	🗴	🗴	🗴	5/10
Logan-Sprenger, 2013 [26]	🗴	🗴	🗴	🗴	🗴	🗴	🗴	🗴	🗴	🗴	4/10
Neufer, 1991 [20]	🗴	🗴	🗴	🗴	🗴	🗴	🗴	🗴	🗴	🗴	3/10
González-Alonso, 1999 [25]	🗴	🗴	🗴	🗴	🗴	🗴	🗴	🗴	🗴	🗴	3/10
Febbraio, 1996 [14]	🗴	🗴	🗴	🗴	🗴	🗴	🗴	🗴	🗴	🗴	4/10
Febbraio, 1996 [16]	🗴	🗴	🗴	🗴	🗴	🗴	🗴	🗴	🗴	🗴	3/10
Febbraio, 1994 [13]	🗴	🗴	🗴	🗴	🗴	🗴	🗴	🗴	🗴	🗴	5/10

1 = Subjects were randomly allocated to groups (in a crossover study, subjects were randomly allocated an order in which treatments were received); 2 = allocation was concealed; 3 = the groups were similar at baseline regarding the most important prognostic indicators; 4 = there was blinding of all subjects; 5 = there was blinding of all therapists who administered the therapy; 6 = there was blinding of all assessors who measured at least one key outcome; 7 = measures of at least one key outcome were obtained from more than 85% of the subjects initially allocated to groups; 8 = all subjects for whom outcome measures were available received the treatment or control condition as allocated or, where this was not the case, data for at least one key outcome were analyzed by “intention to treat”; 9 = the results of between-group statistical comparisons were reported for at least one key outcome; 10 = the study provides both point measures and measures of variability for at least one key outcome [21]; 🗴 = Yes; 🗴 = No.

## References

[1] McArdle D.W., Katch I.F., Katch L.V. (2010). Exercise Physiology. Nutrition, Energy, and Human Performance.

[2] Olsson B.Y.K.E., Saltin B. (1970). Variation in Total Body Water with Muscle Glycogen Changes in Man. Acta Physiol. Scand..

[3] Fernández-Elías V.E., Ortega J.F., Nelson R.K., Mora-Rodriguez R. (2015). Relationship between muscle water and glycogen recovery after prolonged exercise in the heat in humans. Eur. J. Appl. Physiol..

[4] Fernández-Elías V.E., Hamouti N., Ortega J.F., Mora-Rodríguez R. (2015). Hyperthermia, but not muscle water deficit, increases glycogen use during intense exercise. Scand. J. Med. Sci Sports..

[5] Mora-Rodríguez R., Sanchez-Roncero A., Fernández-Elías V.E., Guadalupe-Grau A., Ortega J.F., Dela F., Helge J.W. (2016). Aerobic exercise training increases muscle water content in obese middle-age men. Med. Sci. Sports Exerc..

[6] Bergström J., Hermansen L., Hultman E., Saltin B. (1967). Diet, Muscle Glycogen and Physical Performance. Acta Physiol. Scand..

[7] Trangmar S.J., González-Alonso J. (2019). Heat, Hydration and the Human Brain, Heart and Skeletal Muscles. Sports Med..

[8] Shiose K., Takahashi H., Yamada Y. (2022). Muscle Glycogen Assessment and Relationship with Body Hydration Status: A Narrative Review. Nutrients.

[9] Hamouti N., Del Coso J., Mora-Rodriguez R. (2013). Comparison between blood and urinary fluid balance indices during dehydrating exercise and the subsequent hypohydration when fluid is not restored. Eur. J. Appl. Physiol..

[10] Mora-Rodríguez R., Fernández-Elías V.E., Hamouti N., Ortega J.F. (2015). Skeletal muscle water and electrolytes following prolonged dehydrating exercise. Scand. J. Med. Sci. Sports.

[11] Haussinger D., Lang F., Gerok W. (1994). Regulation of cell function by the cellular hydration state. Am. J. Physiol..

[12] Häussinger D. (1996). The role of cellular hydration in the regulation of cell function. Biochem. J..

[13] Febbraio M.A., Snow R.J., Stathis C.G., Hargreaves M., Carey M.F. (1994). Effect of heat stress on muscle energy metabolism during exercise. J. Appl. Physiol..

[14] Febbraio M.A., Snow R.J., Stathis C.G., Hargreaves M., Carey M.F. (1996). Blunting the rise in body temperature reduces muscle glycogenolysis during exercise in humans. Exp. Physiol..

[15] Hargreaves M., Dillo P., Angus D., Febbraio M. (1996). Effect of fluid ingestion on muscle metabolism during prolonged exercise. J. Appl. Physiol..

[16] Febbraio M.A., Carey M.F., Snow R.J., Stathis C.G., Hargreaves M. (1996). Influence of elevated muscle temperature on metabolism during intense, dynamic exercise. Am. J. Physiol. Integr. Comp. Physiol..

[17] Ivy J.L., Katz A.L., Cutler C.L., Sherman W.M., Coyle E.F. (1988). Muscle glycogen synthesis after exercise: Effect of time of carbohydrate ingestion. J. Appl. Physiol..

[18] Ivy J.L., Lee M.C., Brozinick J.T., Reed M.J. (1988). Muscle glycogen storage after different amounts of carbohydrate ingestion. J. Appl. Physiol..

[19] Jentjens R., Jeukendrup A.E. (2003). Determinants of post-exercise glycogen synthesis during short-term recovery. Sports Med..

[20] Neufer P.D., Sawka M.N., Young A.J., Quigley M.D., Latzka W.A., Levine L. (1991). Hypohydration does not impair skeletal muscle glycogen resynthesis after exercise. J. Appl. Physiol..

[21] Cashin A.G., McAuley J.H. (2020). Clinimetrics: Physiotherapy Evidence Database (PEDro) Scale. J. Physiother..

[22] Viechtbauer W. (2010). Conducting Meta-Analyses in R with the Metafor Package. JSS J. Stat. Softw..

[23] Cochran W.G. (1954). The Combination of Estimates from Different Experiments. Biometrics.

[24] Jamovi (2021). The Jamovi Project [Computer Software]. https://www.jamovi.org.

[25] González-Alonso J., Calbet J.A.L., Nielsen B. (1999). Metabolic and thermodynamic responses to dehydration-induced reductions in muscle blood flow in exercising humans. J. Physiol..

[26] Logan-Sprenger H.M., Heigenhauser G.J.F., Jones G.L., Spriet L.L. (2013). Increase in Skeletal-Muscle Glycogenolysis and Perceived Exertion With Progressive Dehydration During Cycling in Hydrated Men. Int. J. Sport Nutr. Exerc. Metab..

[27] Costill D.L., Cote R., Fink W. (1976). Muscle water and electrolytes following varied levels of dehydration in man. J. Appl. Physiol..

[28] Palmer M.S., Heigenhauser G., Duong M., Spriet L.L. (2017). Ingesting A Sports Drink Enhances Simulated Ice Hockey Performance While Reducing Perceived Effort. Int. J. Sports Med..

[29] Palmer M.S., Heigenhauser G.J.F., Duong M., Spriet L.L. (2017). Mild dehydration does not influence performance or skeletal muscle metabolism during simulated ice hockey exercise in men. Int. J. Sport Nutr. Exerc. Metab..

[30] Burge C.M., Carey M.F., Payne W.R. (1993). Rowing performance, fluid balance, and metabolic function following dehydration and rehydration. Med. Sci. Sports Exerc..

[31] Logan-Sprenger H.M., Heigenhauser G.J.F., Killian K.J., Spriet L.L. (2012). Effects of dehydration during cycling on skeletal muscle metabolism in females. Med. Sci. Sports Exerc..

[32] Sawka M.N., Burke L.M., Eichner E.R., Maughan R.J., Montain S.J., Stachenfeld N.S. (2007). Exercise and fluid replacement. Med. Sci. Sports Exerc..

